# Mitochondrial DNA Affects the Expression of Nuclear Genes Involved in Immune and Stress Responses in a Breast Cancer Model

**DOI:** 10.3389/fphys.2020.543962

**Published:** 2020-11-24

**Authors:** Carole Grasso, David A. Eccles, Stepana Boukalova, Marie-Sophie Fabre, Rebecca H. Dawson, Jiri Neuzil, Patries M. Herst, Michael V. Berridge

**Affiliations:** ^1^Malaghan Institute of Medical Research, Wellington, New Zealand; ^2^Institute of Biotechnology, Czech Academy of Sciences, Vestec, Czechia; ^3^School of Medical Science, Griffith University, Southport, QLD, Australia; ^4^Department of Radiation Therapy, University of Otago, Wellington, New Zealand

**Keywords:** mitochondrial DNA, gene expression, breast cancer, 4T1 model, tumor macrophages

## Abstract

Tumor cells without mitochondrial (mt) DNA (ρ^0^ cells) are auxotrophic for uridine, and their growth is supported by pyruvate. While ATP synthesis in ρ^0^ cells relies on glycolysis, they fail to form tumors unless they acquire mitochondria from stromal cells. Mitochondrial acquisition restores respiration that is essential for *de novo* pyrimidine biosynthesis and for mitochondrial ATP production. The physiological processes that underpin intercellular mitochondrial transfer to tumor cells lacking mtDNA and the metabolic remodeling and restored tumorigenic properties of cells that acquire mitochondria are not well understood. Here, we investigated the changes in mitochondrial and nuclear gene expression that accompany mtDNA deletion and acquisition in metastatic murine 4T1 breast cancer cells. Loss of mitochondrial gene expression in 4T1ρ^0^ cells was restored in cells recovered from subcutaneous tumors that grew from 4T1ρ^0^ cells following acquisition of mtDNA from host cells. In contrast, the expression of most nuclear genes that encode respiratory complex subunits and mitochondrial ribosomal subunits was not greatly affected by loss of mtDNA, indicating ineffective mitochondria-to-nucleus communication systems for these nuclear genes. Further, analysis of nuclear genes whose expression was compromised in 4T1ρ^0^ cells showed that immune- and stress-related genes were the most highly differentially expressed, representing over 70% of those with greater than 16-fold higher expression in 4T1 compared with 4T1ρ^0^ cells. The monocyte recruiting chemokine, Ccl2, and Psmb8, a subunit of the immunoproteasome that generates MHCI-binding peptides, were the most highly differentially expressed. Early monocyte/macrophage recruitment into the tumor mass was compromised in 4T1ρ^0^ cells but recovered before mtDNA could be detected. Taken together, our results show that mitochondrial acquisition by tumor cells without mtDNA results in bioenergetic remodeling and re-expression of genes involved in immune function and stress adaptation.

## Introduction

Intercellular mitochondrial transfer is a recently discovered phenomenon that has been shown to occur both *in vitro* (Spees et al., 2006; Berridge et al., 2016) and *in vivo* (Islam et al., 2012; Ahmad et al., 2014; Tan et al., 2015; Hayakawa et al., 2016; Osswald et al., 2016; Dong et al., 2017; Marlein et al., 2017, 2019). Respiration-deficient tumor cell lines without mitochondrial (mt) DNA (ρ^0^ cells), obtained by long-term exposure to low-dose ethidium bromide (King and Attardi, 1989), were shown to acquire mtDNA through intercellular mitochondrial transfer following co-culture with respiration-competent donor cells (Spees et al., 2006). Mitochondrial acquisition from stromal cells by metastatic breast cancer (4T1) and melanoma (B16) ρ^0^ cells injected into syngeneic mice was also observed with complete restoration of respiration and tumorigenicity (Tan et al., 2015; Dong et al., 2017).

Cells without mtDNA lack key subunits of mitochondrial respiratory chain complexes I, III, and IV, as well as two structural subunits of complex V that facilitate ATP synthase activity. They have no mitochondrial electron transport, cannot generate ATP through oxidative phosphorylation (OXPHOS), and rely exclusively on glycolysis for ATP production. Nevertheless, they maintain the mitochondrial membrane potential required for protein import into the mitochondria *via* the adenine nucleotide carrier and ATPase activity of sub-complex V (Appleby et al., 1999). Cells lacking mtDNA are auxotrophic for uridine, and most require additional pyruvate *in vitro*, a substrate for lactate dehydrogenase that re-oxidizes NADH produced during glycolysis and in the TCA cycle (Larm et al., 1994). In respiration-competent cells, most NADH is oxidized during mitochondrial respiration (see Herst et al., 2018b). However, uridine is essential because *de novo* pyrimidine biosynthesis is compromised in non-respiring ρ^0^ cells. These cells are unable to re-oxidize ubiquinol to ubiquinone, an essential electron acceptor not only for respiratory complexes I and II but also for dihydroorotate dehydrogenase (DHODH). Located at the outer surface of the inner mitochondrial membrane, DHODH catalyzes the fourth step in the *de novo* pyrimidine biosynthetic pathway (Evans and Guy, 2004). The importance of DHODH activity for tumor formation was shown by the inability of 4T1 breast carcinoma and B16 melanoma cells lacking the *Dhodh* gene to form tumors, with restoration of tumor formation after re-expression of the gene (Bajzikova et al., 2019).

When ρ^0^ tumor cells are injected into mice, they are immediately deprived of uridine. In order to form tumors, these cells acquire mitochondria from donor cells in the host to restore respiration and DHODH activity, allowing them to synthesize nucleic acids and divide. Although cells capable of donating intact mitochondria have been identified *in vitro* (Berridge et al., 2015; Herst et al., 2018a; Berridge et al., 2020), the physiologically relevant donor cells in different tissues are not known, and the signaling mechanisms that mediate intercellular mitochondrial transfer remain unclear. To better understand the cellular and molecular processes involved in mitochondrial transfer between cells, we investigated transcriptional differences between 4T1 and 4T1ρ^0^ cells and cells derived from subcutaneous tumors that grew from 4T1ρ^0^ cells (4T1ρ^0^ SC) using unbiased MinION long-read cDNA sequencing.

Differential gene expression between human tumor cell lines with and without mtDNA has been investigated previously by serial analysis of gene expression (SAGE) and quantitative real-time PCR using human 143B osteosarcoma cells (Duborjal et al., 2002). A set of nine nuclear genes encoding mitochondrial OXPHOS complex subunits was shown to be unchanged in 143Bρ^0^ cells. In addition, microarray approaches with MBA-MB-435 breast carcinoma cells (Delsite et al., 2002), A459 lung adenocarcinoma cells (Magda et al., 2008), and 143B and A459 cells (Mineri et al., 2009) reported both up- and down-regulation of the expression of nuclear genes between cells with and without mtDNA. Changes involved a variety of cell properties including cell cycle regulation and growth, cell signaling, respiration, and energy metabolism.

We have recently investigated the relative expression of selected nuclear genes in 4T1, 4T1ρ^0^, and derived cell lines following mitochondrial acquisition (Tan et al., 2015) and B16, B16ρ^0^, and derived cell lines with acquired mitochondria (Dong et al., 2017) by quantitative PCR (qPCR). While the lower expression of nuclear-encoded *Sdha*, *b*, and *d* and selected complex I, III, IV, and V genes of up to 70% relative to parental cells was observed in 4T1ρ^0^ cells and in 4T1ρ^0^ SC cells (Tan et al., 2015), similar effects were not observed in B16ρ^0^ cells or B16ρ^0^ SC cells (Dong et al., 2017), suggesting that these effects may be tumor specific. In another study, we investigated the gene expression in 4T1, 4T1ρ^0^, and derived cell lines isolated at different times following injection using an array of genes encoding proteins that are imported into the mitochondria (Bajzikova et al., 2019). Patterns of gene expression clustered around the level of respiration recovery.

Because the scope of these approaches was constrained and designed to address specific questions, we decided to investigate the gene expression in the syngeneic 4T1 breast cancer model using cell lines with and without mtDNA and following mitochondrial acquisition. The aim was to better understand the role of mtDNA in the physiological processes involved in intercellular mitochondrial transfer, respiration recovery, and subsequent metabolic remodeling. Here, we show that most nuclear genes encoding mitochondrial respiratory complex subunits and mitochondrial ribosomal proteins were not markedly affected by the absence of mtDNA. The most highly differentially expressed, not expressed or poorly expressed in 4T1ρ^0^ cells, were associated with immune responses and stress adaptation rather than metabolic remodeling.

## Materials and Methods

### Cell Culture, Tumor Formation, and Establishment of Tumor-Derived Cells

Full details of cell culture, tumor formation, and establishment of tumor-derived cells can be found in previously published work (Tan et al., 2015). Briefly, 4T1 cells sourced from ATCC were grown in Nunc flasks in RPMI 1640 medium containing 10% fetal bovine serum (Gibco), 2 mM GlutaMAX, and 100 U/mL penicillin and 100 μg/ml streptomycin at 37°C in a 5% CO_2_ incubator. 4T1ρ^0^ cells were supplemented with 1 mM pyruvate and 50 μg/ml uridine. All consumables were purchased from Thermo Fisher Scientific (New Zealand) unless stated otherwise. For the preparation of 4T1ρ^0^ cells, parental tumor cell lines were cultured for 10–12 weeks in low-dose ethidium bromide (50–100 ng/ml) supplemented with 1 mM pyruvate and 50 μg/ml uridine, followed by transfer to medium lacking ethidium bromide. Loss of mtDNA was monitored by sensitivity to 2 μM FCCP, PCR analysis loss of mitochondrial *Cytb* gene, and by pyruvate/uridine auxotrophy.

4T1 and 4T1ρ^0^ cells (10^5^) were injected subcutaneously into the right flank of female Balb/c mice. 4T1ρ^0^ sublines were derived from primary subcutaneous tumors (4T1ρ^0^ SC) and cultured in the presence of 60 μM 6-thioguanine (6-TG) for 7–10 days. The 6-TG-resistant tumor cells survived and grew in culture. Mitochondrial acquisition by 4T1ρ^0^ SC cells was established using transmission electron microscopy (TEM) and sequencing (Tan et al., 2015), confirming the presence of the host’s mitochondrial-specific single nucleotide polymorphism at position 16076 in the D-Loop and polyA region in location 9821.

### MinION cDNA Sequencing and Mapping

RNA from 4T1 and 4T1ρ^0^ cells was extracted (Qiagen RNeasy Mini Kit) and quantified using the NanoDrop One Spectrophotometer (Thermo Fisher Scientific). RNA (50 ng) was used for library preparation for Oxford Nanopore Technologies (ONT) long-read cDNA sequencing. Samples were processed according to the most up-to-date ONT cDNA rapid barcoding protocol at the time of sequencing (SQK-PCS108, SQK-PCB109 with SQK-PBK004). Briefly, this process involved reverse transcription of extracted RNA using a custom poly TVN primer that binds to polyA RNA sequences, second-strand synthesis using a custom strand-switch primer, followed by PCR to incorporate barcode rapid attachment primers, to which rapid adapters were bound. For sequencing, libraries were prepared with up to six cDNA samples and on nine R9.4.1 ONT flow cells.

### Data Analysis of MinION Reads

The program LAST was used to identify ONT barcodes present in sequenced reads, followed by a customized program (Eccles, 2019), designed to use barcode assignments to demultiplex reads into files based on their incorporated barcodes. Demultiplexed reads were then mapped to the ONT strand-switch primer sequence in order to identify the direction of transcription. Reads were mapped to the mouse transcriptome using LAST, grouped by mapped transcript, and counted producing a table that was further processed using DESeq2 (Love et al., 2014) to determine the differential expression (log_2_ fold change) between 4T1 and 4T1ρ^0^ cell lines and between 4T1 and 4T1ρ^0^ SC cells. Standard error of the mean (SEM) log_2_ fold change was estimated as the square root of the mean squared standard error of each calculated log_2_ fold change as reported by DESeq2. We used FDR-adjusted *p* values reported by DESeq2 (Benjamini and Hockberg, 1995) of 0.1 as a threshold for statistical significance. Genes were categorized as stress- or immune-related based on descriptions provided in GeneCards and validated using the protein–protein interaction network database, String.

### qPCR

Primers specific for individual genes were designed using the NCBI–NIH primer designing tool Primer-BLAST. These were: *Ccl2* (CAGGTCCCTGTCATGCTTCT and GAGTGGGG CGTTAACTGCAT), *Psmb8* (ACTACAGTTTCTCCGCGCAA and TTGAAGGCGAGTGTGGTTGT), *Sumo3* (GATGGCT CGGTGGTACAGTT and ACCGGAATCGAATCTGCCTC), *Ccl5* (GTGCCCACGTCAAGGAGTATT and CTTGGCGGTT CCTTCGAGT), Gng11 (CGCAAAGAAGTCAAGTTGCAGA and CTGGGATTCCCTTTACCAGAGG), *Serpinf1* (ACGATA CGGCTTGGACTCTG and TCAAGTTCTGGGTCACGGTC), *Gstk1* (CGTGTATGGTCTCGAGATGAAGAT and CAGA AAGTGTTGGGCTTGCG), and *Cst6* (GCGACAGCCTCTACT ACTTCC and GTCTTTCGGCACTCTGTGCT). *Rplp0* (TAACC CTGAAGTGCTCGACAT and GTACCCGATCTGCAGACA CAC), and *Ppia* (ACGCCACTGTCGCTTTTC and CTGCA AACAGCTCGAAGGA) were used as housekeeping genes.

RNA was isolated from 4T1, 4T1ρ^0^, and 4T1ρ^0^ SC cells using Quick-RNA Miniprep (Zymo Research, New Zealand), and cDNA was obtained with SuperScript IV Reverse Transcriptase (Thermo Fisher Scientific, New Zealand). qPCR was then performed on an ABI7300 thermocycler (QuantStudio) using SYBR Green Master Mix (Applied Biosystems) with 5 ng/4 μl cDNA per reaction on fast cycling mode.

### mtDNA Sequencing

4T1 and 4T1ρ^0^ cells at 10^6^ per animal were injected subcutaneously into the flank of Balb/c female mice. The animals were sacrificed, and the pre-tumor regions were removed on Days 1, 2, and 3. The tissues were dissociated and passed through a 40 μM filter into a single-cell suspension and grown in medium containing uridine, pyruvate, and 60 μM 6-TG to obtain pure cultures of 4T1 and 4T1ρ^0^ SC cells. DNA was extracted using Qiagen Blood & Tissue Kit and quantified on NanoDrop One Spectrophotometer. For mtDNA sequencing, the forward primer (5′ TCA GTA CTT CTA GCA TCA GGT GT 3′) and reverse primer (5′ GCT AGG CAG AAT AGG AGT GAT G 3′) were used to amplify a 2,149 bp fragment located specifically in the mitochondrial genome. PCR products were analyzed by gel electrophoresis on a 2% agarose gel and sequenced using the Sanger method.

### Confocal Microscopy

Tumor tissue was snap frozen; 10 μM sections were fixed in acetone, permeabilized, and non-specific staining blocked. For Day 1 tissue, macrophages were visualized by co-staining with Alexa Fluor 647 anti-mouse F4/80 (BioLegend), CellTrace Violet, and anti-mouse fibroblast activation protein-α (FAP) (Thermo Fisher Scientific). Images were captured *via* confocal microscopy (Olympus FV12000). For calculation of macrophage infiltration, cells were stained with α-F4/80 (macrophage/red) and CellTrace Violet (tumor cells/blue). FIJI software was used to calculate the % of red pixels (macrophages)/% of blue pixels (tumor cells). At least 50 images were taken for each tumor, and 4–5 tumors were analyzed for each time point. The % of red pixels/% of blue pixels of each photograph per time point is shown in box plots. The boxes represent the interquartile range (25–75%) with a line at the median and whiskers representing the minimum and maximum values.

## Results

**FIGURE 1:**
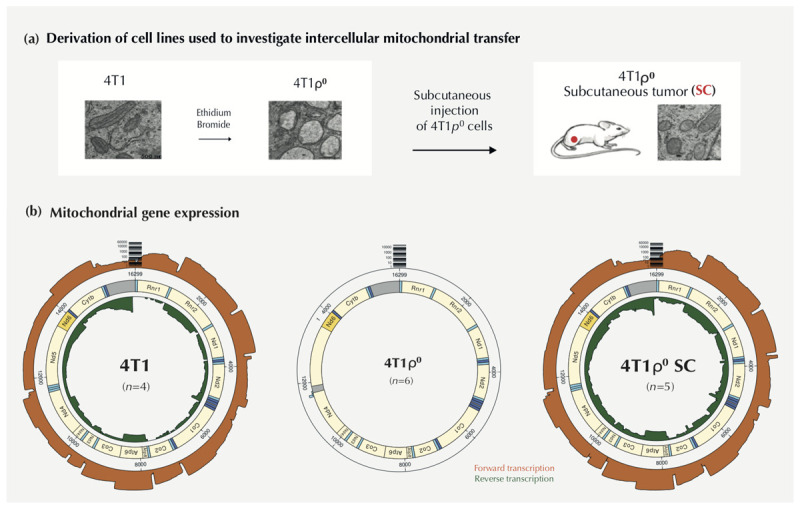
4T1 cell lines used to investigate differential gene expression following mtDNA deletion and acquisition. **(a)** Diagrammatic representation of 4T1 cell lines used. 4T1 cells were exposed to ethidium bromide for approximately 12 weeks to deplete cells of mtDNA (4T1ρ^0^ cells). Mitochondrial morphology (see Tan et al., 2015) appears swollen in 4T1ρ^0^ cells with few internal membrane structures. 1 × 10^5^ 4T1ρ^0^ cells were injected subcutaneously (SC) into female Balb/c mice, and tumors were excised at Day 28. Cells resistant to 6-thioguanine in culture were isolated to derive a 4T1ρ^0^ SC cell line. 4T1ρ^0^ SC cell line showed mitochondrial morphology similar to the parental cell line following mitochondrial acquisition from the host. **(b)** The left image gives a radial depiction of transcripts of mitochondrially encoded genes (*n* = 4) using MinION cDNA long-read sequencing with forward transcription (orange) and reverse transcription (green). The middle image shows the absence of mitochondrial gene expression in 4T1ρ^0^ cells (*n* = 3). The right image shows the complete restoration of the mitochondrial gene expression in 4T1ρ^0^ SC cells (*n* = 5).

To investigate the transcriptional changes that occur in cells without mtDNA and following mitochondrial acquisition, we used unbiased MinION long-read cDNA sequencing of transcripts from 4T1 cells, 4T1ρ^0^ cells, and a cell line derived on Day 28 from a subcutaneous tumor that grew from 4T1ρ^0^ cells (4T1ρ^0^ SC) (Tan et al., 2015). As shown diagrammatically in Figure 1a, the mitochondrial morphology of 4T1ρ^0^ cells was distended with low density internal staining and extensive loss of cristae compared with parental 4T1 cells (see Tan et al., 2015). 4T1ρ^0^ SC cells showed partial recovery of parental mitochondrial internal structures.

### Effects of mtDNA Deletion and Acquisition on Mitochondrial Gene Expression

The effects of mtDNA deletion and acquisition on forward (heavy chain, orange) and reverse (light chain, green) transcription of the circular mitochondrial genome of 4T1, 4T1ρ^0^, and 4T1ρ^0^ SC cells are shown in Figure 1b. We observed a small amount of multiplex spillover in nanopore cDNA sequencing runs (about 0.1%), as evidenced by the presence of a very small number of mitochondrial reads associated with 4T1ρ^0^ barcodes when sequenced together with 4T1 containing mtDNA (Supplementary Figure 1). No mitochondrial transcripts were found when 4T1ρ^0^ cell lines were multiplexed and sequenced together without other cell lines (Figure 1b).

Complete loss of mitochondrial transcripts in 4T1ρ^0^ cells was restored in 4T1ρ^0^ SC cells with the pattern of gene expression being almost identical to that with 4T1 cells. The results also show that the expression of individual mitochondrial genes in 4T1 and 4T1ρ^0^ SC cells varies by more than two orders of magnitude, consistent with steady-state transcriptional control previously demonstrated for the human mitochondrial genome (Duborjal et al., 2002).

### Effects of mtDNA Deletion and Acquisition on the Expression of Nuclear Genes Encoding Mitochondrial OXPHOS Subunits and Mitochondrial Ribosomal Subunits

**FIGURE 2:**
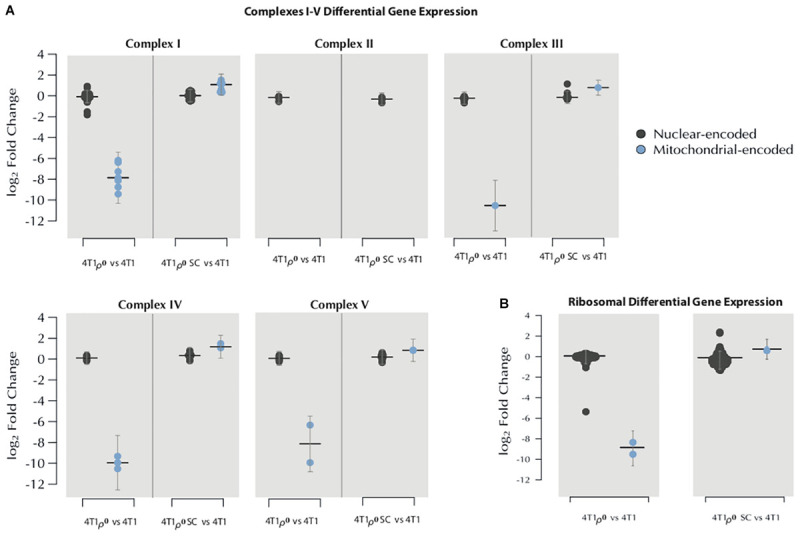
Effects of mtDNA deletion and acquisition on the expression of nuclear and mitochondrial genes encoding mitochondrial OXPHOS complexes and mitochondrial ribosomal subunits. **(A)** MinION long-read cDNA sequencing was used to determine the log_2_ fold change in the differential gene expression of OXPHOS subunits complexes I–V, comparing 4T1ρ^0^ cells without mtDNA (*n* = 3) with 4T1 parental cells (*n* = 4) (left panel of each graph) and 4T1ρ^0^ SC cells with acquired mitochondria (*n* = 5) and 4T1 cells (right panel of each graph). Blue: mitochondrial-encoded genes for CI (seven subunits), CIII (one subunit), CIV (three subunits), and CV (two subunits). Black: 72 nuclear genes encoding subunits of complexes. **(B)** log_2_ fold change of the differential gene expression of nuclear-encoded mitochondrial ribosomal subunits (left panel of each comparative graph) and mitochondrially encoded rRNA (right panel of each comparative graph).

We next looked at how the expression of nuclear genes encoding subunits of mitochondrial respiratory complexes, CI–CIV, and ATP synthase, CV, was affected by mtDNA deletion in 4T1ρ^0^ cells. Figure 2A (left panel of each set of comparisons) compares the expression of the 13 mitochondrial genes that encode subunits of CI (seven subunits), CIII (one subunit), CIV (three subunits), and CV (two subunits), with the 72 nuclear genes that encode subunits of these complexes. In this analysis, we included CII as an additional control, where all four subunits are nuclear-encoded. In sharp contrast to the loss of mitochondrial gene expression in 4T1ρ^0^ cells, the expression of the 76 nuclear genes encoding OXPHOS subunits was on average similar between 4T1 and 4T1ρ^0^ cells.

Nuclear and mitochondrial transcripts for CI–CV subunits were then compared between 4T1 and 4T1ρ^0^ SC cells following mitochondrial acquisition. Figure 2A (right panel of each set of comparisons) shows that 4T1ρ^0^ SC cells fully recovered mitochondrial gene expression for subunits of CI, CIII, CIV, and CV, whereas the expression of nuclear genes encoding subunits of these complexes remained similar between 4T1, 4T1ρ^0^, and 4T1ρ^0^ SC cells. A similar analysis comparing nuclear transcript levels of the 12S and 16S mitochondrial ribosomal RNA showed few marked changes in the expression of nuclear genes encoding mitochondrial ribosomal subunits between 4T1, 4T1ρ^0^ cells, and 4T1ρ^0^ SC cells that had acquired mitochondria (Figure 2B).

### Effects of mtDNA Deletion and Acquisition on the Expression of Nuclear Genes Not Involved in Mitochondrial OXPHOS Complexes or Mitochondrial Ribosome Formation

**FIGURE 3:**
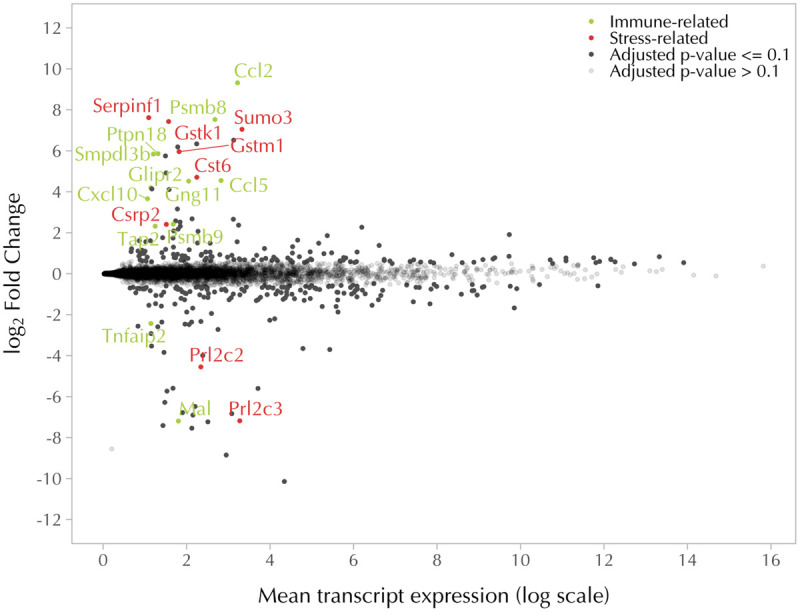
mtDNA deletion affects the expression of nuclear genes involved in immune- and stress-related functions. MA scatter plot showing log_2_ fold change and mean transcript expression of nuclear genes differentially expressed in 4T1 (*n* = 4) and 4T1ρ^0^ cells (*n* = 3). Nuclear genes encoding respiratory complex subunits and mitochondrial ribosomal subunits were excluded. Genes with an adjusted log_2_ fold change in *p-*value of ≤0.1 were expressed higher in 4T1 (top) and higher in 4T1ρ^0^ (bottom) in black, unless immune-related (green dots) or stress-related (red dots). Genes with a *p*-value of ≥0.1 are in gray.

To better understand the effects of mtDNA on nuclear gene expression, we compared the expression of nuclear genes not involved in OXPHOS complexes and mitochondrial ribosome formation between 4T1 and 4T1ρ^0^ cells. After adjusting for read variation between sequencing runs, differential gene expression was visualized in a modified Bland–Altman MA plot adjusting for high variance at low gene expression levels (Figure 3). First, we focused on genes whose expression was compromised in 4T1ρ^0^ cells.

#### Differentially Expressed Nuclear Genes With Little or No Expression in 4T1ρ^0^ Cells

**FIGURE 4:**
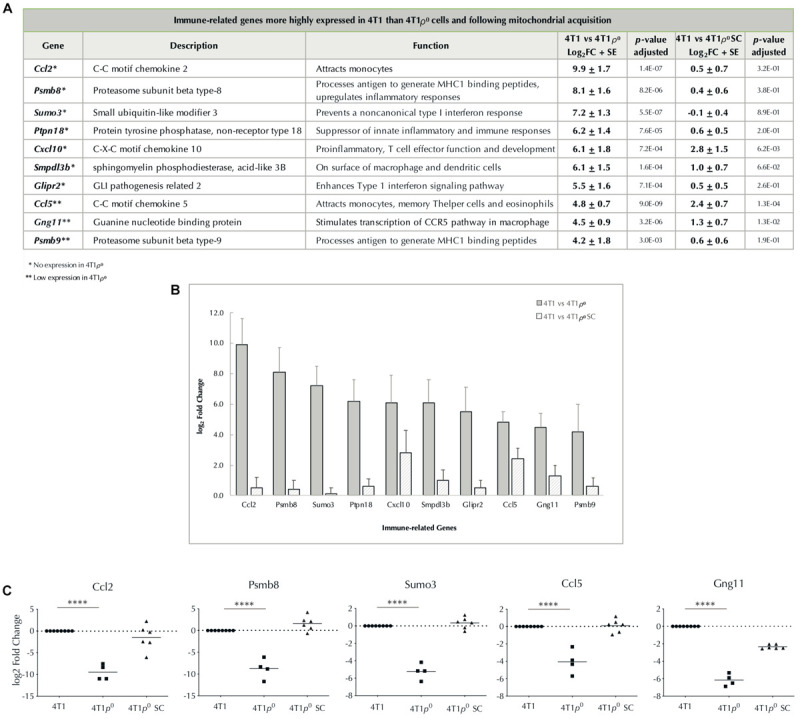
Expression of nuclear-encoded immune-related genes absent/low in cells deleted of mtDNA and restored/partially restored after cells acquire mitochondria. **(A)** Table of immune-related genes with log_2_ fold change >4, higher in 4T1 cells than in 4T1ρ^0^ cells, and 4T1 cells than in 4T1ρ^0^ SC cells that have acquired mitochondria. Standard error (SE) is based on average log_2_ fold change and *p*-value adjusted to account for false discovery rate. Genes not expressed in 4T1ρ^0^ (^∗^). Genes weakly expressed in 4T1ρ^0^ (^∗∗^). **(B)** Bar graph of **(A)** showing log_2_ fold change of immune-related genes higher in 4T1 cells than in 4T1ρ^0^ and 4T1ρ^0^ SC cells. **(C)** log_2_ fold change in the expression of immune-related genes *Ccl2*, *Psmb8*, *Sumo3*, *Ccl5*, and *Gng11* from table **(A)** validated by qPCR in 4T1, 4T1ρ^0^, and 4T1ρ^0^ SC cells. *p*-Values were determined from Ct values prior to log transformation and normalization; ^****^p < 0.0001.

The two most highly differentially expressed transcripts expressed in 4T1 cells but not 4T1ρ^0^ cells were the immune response-related genes, *Ccl2* and *Psmb8* (Figures 4A,B). In addition, another 8 immune-related genes were among the 21 most highly differentially expressed genes with little or no expression in 4T1ρ^0^ cells. Of these, *Ccl5* and *Psmb9* are closely functionally related to *Ccl2* and *Psmb8*, respectively. Ccl2, Ccl5, and Cxcl10 are associated with inflammatory immune responses involving monocyte/macrophage recruitment, whereas Psmb8 and Psmb9 are involved in phenotype switching of macrophages to facilitate a type 2 tumor-promoting cytokine environment (Chen et al., 2016). The expression of a selection of these genes was validated by qPCR in 4T1, 4T1ρ^0^, and 4T1ρ^0^ SC cells (Figure 4C). Differential expression of these genes between 4T1 and 4T1ρ^0^ cells was confirmed. While the expression of *Ccl2*, *Psmb8*, and *Ccl5* recovered fully in 4T1ρ^0^ SC cells, *Gng11* that is involved in the transcriptional upregulation of the Ccl5 pathway in macrophage recruitment (Noerholm et al., 2011) recovered only partially.

**FIGURE 5:**
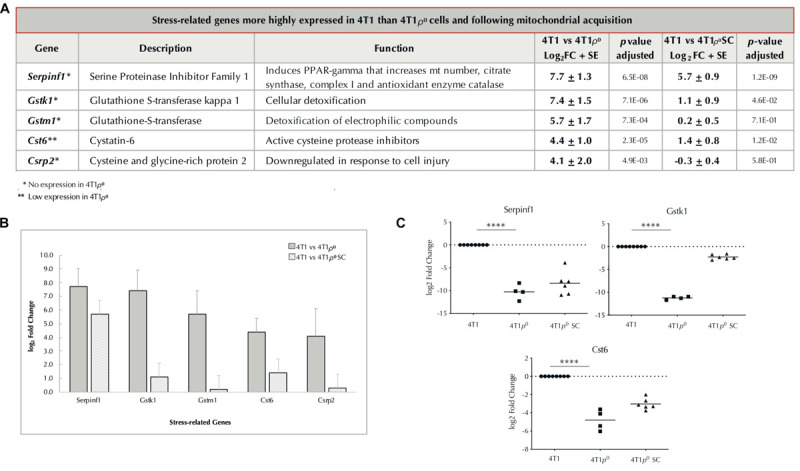
Expression of nuclear-encoded stress-related genes absent/low in cells without mtDNA and restored/partially restored after cells acquire mitochondria. **(A)** Table of stress-related genes with log_2_ fold change >4, higher in 4T1 cells than in 4T1ρ^0^ cells, and in 4T1 cells than in 4T1ρ^0^ SC cells that have acquired mitochondria. Standard error (SE) based on average log_2_ fold change. *p*-Value adjusted to account for false discovery rate. Genes not expressed in 4T1ρ^0^ (^∗^). Genes weakly expressed in 4T1ρ^0^ (^∗∗^). **(B)** Bar graph of **(A)** showing log_2_ fold change of stress-related genes in 4T1 cells compared with 4T1ρ^0^ and 4T1ρ^0^ SC cells. **(C)** log_2_ fold change in the expression of stress-related genes *Serpinf1*, *Gstk1*, and *Cst6* from table **(A)** validated by qPCR in 4T1, 4T1ρ^0^, and 4T1ρ^0^ SC cells. *p*-Values were determined from Ct values prior to log_2_ transformation and normalization; ^****^p < 0.0001.

Another group of five highly differentially expressed genes in the top 21 whose expression was compromised in 4T1ρ^0^ cells was stress-related genes (Figures 5A,B). Of these, *Serpinf1* and *Gstk1* were the most highly differentially expressed. Differential expression of these two stress-related genes and *Cst6* was confirmed by qPCR (Figure 5C). Recovery of the expression of stress-related genes in 4T1ρ^0^ SC cells was more variable than that of immune-related genes, and this variability was confirmed by qPCR with the genes tested.

While immune- and stress response-related genes were the five most highly differentially expressed (4T1 > 4T1ρ^0^) and comprised 15 of the top 21 most differentially expressed genes in this group, six more genes showed at least 16-fold higher expression in 4T1 cells than in 4T1ρ^0^ cells. These genes had diverse functions and were not easily grouped, and none were identified as genes that might be involved in metabolic remodeling (Supplementary Table 1).

All but 4 of the 21 most highly differentially expressed genes whose expression was compromised in 4T1ρ^0^ cells (81%) had no reads indicating that the presence or absence of mtDNA in itself is a critical factor in the expression of this subset of nuclear genes. For those genes whose expression was fourfold higher in 4T1 cells than in 4T1ρ^0^ cells, 29 of 40 genes (73%) showed undetectable reads in 4T1ρ^0^ cells (Supplementary Table 1).

#### Differentially Expressed Nuclear Genes With Higher Expression in 4T1ρ^0^ Than in 4T1 Cells

In the group of genes with more than fourfold higher expression in 4T1ρ^0^ cells than in 4T1 cells, there were only two genes (*Mal* and *Tnfaip2*) that could be linked to immune responses and two genes (*Prl2c2* and *Prl2c3*) involved in cellular responses to nutrient depletion. Surprisingly, genes involved in metabolic remodeling toward glycolysis were not part of this group (Supplementary Table 2).

### Effects of mtDNA Deletion and Acquisition on Macrophage Recruitment Into Early 4T1ρ^0^ Tumors

**FIGURE 6:**
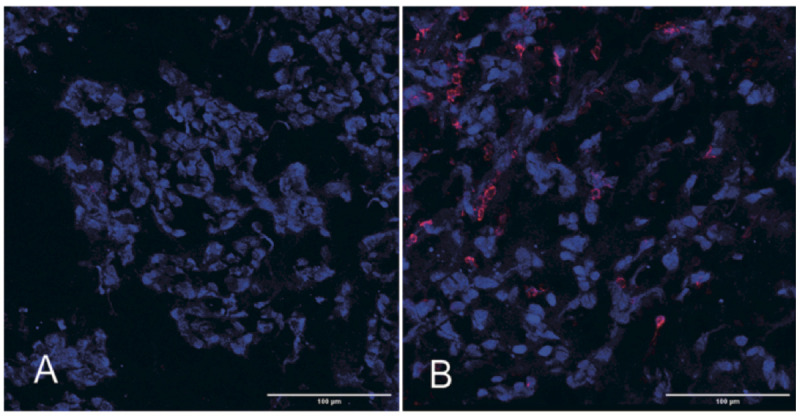
Macrophages are absent in the tumor bulk 24 h after subcutaneous injection with 4T1ρ^0^ cells. Representative confocal images of and 4T1ρ^0^ **(A)** and 4T1 **(B)** tumor tissues from Day 1 tumors stained with the macrophage antibody F4/80 (red) and CellTrace Violet (blue). *n* = 4–5.

Lack of *Ccl2, Ccl5*, and *Cxcl10* expression in 4T1ρ^0^ cells posed the question of whether or not macrophage recruitment into 4T1ρ^0^ tumors early in their development was compromised, and if so, whether or not this affected tumor formation. Initial studies using fluorescence confocal microscopy of cryo-sliced sections stained with anti-F4/80 for macrophages and CellTrace Violet for tumor cells showed a distinct lack of macrophage infiltration into developing tumors 24 h after injection with 4T1ρ^0^ cells (Figure 6A). In contrast, macrophage recruitment into developing 4T1 tumors was clearly evident at this time point (Figure 6B).

**FIGURE 7:**
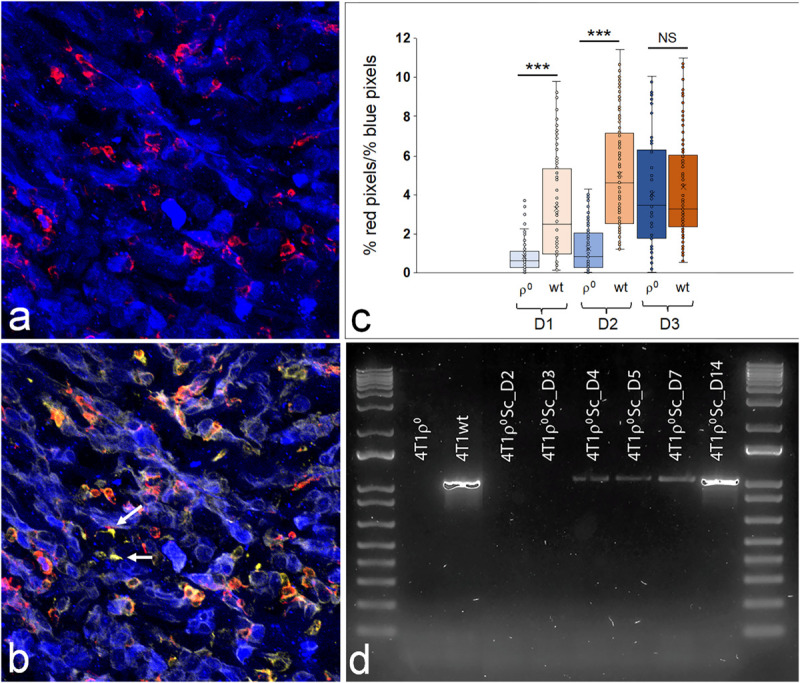
Macrophage infiltration into early 4T1 and 4T1ρ^0^ subcutaneous tumors. Representative confocal image through a 4T1ρ^0^ SC tumor on Day 3 **(a,b)**. Macrophages are red (α-F4/80), and tumor cells are blue (CellTrace Violet) **(a)**. These same macrophages are orange in **(b)**, demonstrating double staining with α-F4/80 (red) and α-FAP (yellow). Cancer-associated fibroblasts are yellow (α-FAP), and tumor cells are blue (celltrace violet). Photographs (*n* = 50 per tumor) were analyzed to determine the level of macrophage infiltration into the bulk of the tumor. Fiji (ImageJ) was used to determine the % of red pixles/% of blue pixles as a measure of macrophage infiltration. A total of 4–5 tumors were analyzed for each time point, with the % of red pixels/% of blue pixels of each photograph per time point shown in box plots. The boxes represent the interquartile range (25–75%) with a line at the median and whiskers representing the minimum and maximum values **(c)**. ***p < 0.001; NS, non-significant (*p* = 0.466). Gel electrophoresis of a mitochondrial 2,149 bp fragment in 4T1, 4T1ρ^0^, and 4T1ρ^0^ SC cells taken from days: D2, D3, D4, D5, D7, and D14 **(d)**.

We next investigated whether or not the lack of macrophage infiltration was sustained and if this affected tumor progression. In order to quantify macrophage infiltration, we collected tumors on different days after injection (*n* = 4–5 per time point), cryo-sliced stained sections through the middle of the tumors, and recorded at least 50 images per tumor using confocal microscopy. Co-staining with anti-F4/80 (macrophage marker) and anti-FAP-α indicated that the majority of macrophages inside the tumor mass were likely of the tumor-permissive M2 phenotype (Figures 7a,b). FIJI (ImageJ) was used to calculate the space taken up by macrophages (% of red pixels) relative to that taken up by tumor cells (% of blue pixels). Macrophage recruitment into the tumor mass was calculated as the ratio of % of red pixels over the % of blue pixels. Figure 7c shows that by Day 3, macrophage recruitment into 4T1ρ^0^ SC tumors had caught up to that in 4T1 tumors.

In order to determine how soon after injection mitochondrial transfer had occurred in the tumors that grew from 4T1ρ^0^ cells, we amplified a 2,149 bp fragment located specifically in the mitochondrial genome using PCR and determined the presence of this fragment by gel electrophoresis (Figure 7d) in subcutaneous 4T1ρ^0^ tumors at different time points. Days 2 and 3 tumors did not contain detectable amounts of mtDNA, but the mtDNA fragment was present from Day 4 onward, reaching parental levels by Day 14.

## Discussion

The ability of tumor cell lines without mtDNA to acquire mitochondria from adjacent cells in the body following inoculation provides a unique opportunity to investigate the adaptive cellular processes involved in intercellular mitochondrial transfer. Mitochondrial transfer restores mitochondrial gene expression, respiration, and mitochondrial ATP production as well as the ability to form tumors (Tan et al., 2015; Dong et al., 2017). While previous investigations have shown relatively small changes in nuclear gene expression in human tumor cell lines with and without mtDNA (Delsite et al., 2002; Magda et al., 2008; Mineri et al., 2009), analytical approaches have been limited to genes of interest or gene sets in commercial microarrays. The focus of these approaches has been on altered metabolic and signaling pathways in human tumor cell lines in culture that are associated with glycolytic adaptation. The use of a tumor cell model that acquires mitochondria *in vivo* has added a layer of potential physiological inquiry not previously available. In addition, we used a relatively new sequencing technique, unbiased long-read MinION nanopore cDNA sequencing in a murine metastatic breast carcinoma model (Tan et al., 2015).

Loss of mitochondrial gene expression in 4T1ρ^0^ cells was shown to be restored *in vivo* across the mitochondrial transcriptome of both the heavy and light strands of the mitochondrial genome. Similar results were obtained with B16ρ^0^ metastatic melanoma cells and with a ρ^0^ cell line derived from immortalized neonatal mouse astrocytes (results not shown). Our results confirm and extend earlier results (Duborjal et al., 2002) by showing that the expression of most of the 76 nuclear-encoding subunits of mitochondrial OXPHOS complexes remains largely unchanged in 4T1ρ^0^ cells. This demonstrates that in the 4T1 model, mitochondrial–nuclear communication is largely ineffective in the absence of mitochondrial transcription. Similar results were obtained with nuclear genes encoding mitochondrial ribosomal proteins. Other authors have shown an increased expression of CoIV and CoVIaL transcripts in human osteosarcoma ρ^0^ cells by Northern blot analysis (Li et al., 1995; Duborjal et al., 2002), whereas Mineri et al. (2009), using microarray and RT-PCR analysis, have reported a small (22–37%) down-regulation of *UQCRFS1* expression in 143B and A549 cells lacking mtDNA and a small (41–65%) up-regulation with the *ATP5D* complex V gene. However, differences in the expression of these nuclear genes were not statistically significant in our analysis of 4T1 and 4T1ρ^0^ cells. Similarly, the small (17–33%) down-regulation of *MRPL20* gene expression observed in 143Bρ^0^ and A549ρ^0^ cells was not evident in our analysis of 4T1 cells with and without mtDNA. Another analysis of nuclear gene expression in human MDA-MB-435 breast carcinoma cells with and without mtDNA showed that *ATP5A1* gene expression was upregulated by more than threefold in ρ^0^ cells (Delsite et al., 2002), but we were unable to detect changes in the expression of this gene between 4T1 and 4T1ρ^0^ cells.

Others have shown variable changes in nuclear-encoded protein subunits of respiratory complexes in human 143B osteosarcoma cells lacking mtDNA (Aretz et al., 2016; He et al., 2017), results that are in line with our limited analysis of nuclear-encoded respiratory complex subunits in the 4T1 model (Tan et al., 2015). The effects of mtDNA deletion on nuclear-encoded respiratory complex subunits were more variable than the changes we observed in steady-state nuclear gene expression of these subunits. Differences between steady-state nuclear gene expression and protein subunit levels of respiratory complexes in ρ^0^ cells compared with parental tumor cells could be explained by post-translational mechanisms.

Expression analysis of nuclear genes not encoding mitochondrial OXPHOS complexes or mitochondrial ribosomal protein subunits showed that 15 of the 22 most highly expressed genes with greater than 16-fold expression in 4T1 cells compared with 4T1ρ^0^ cells could be linked to immune and stress responses. Of these, *Ccl2*, encoding a monocyte/macrophage-recruiting chemokine, and *Psmb8*, encoding a subunit of the immunoproteasome that facilitates protein processing to generate MHCI-binding peptides, were the two most highly differentially expressed genes. Genes encoding Ccl5, another monocyte attracting chemokine, and Psmb9 that has similar function to Psmb8 were also differentially expressed as was the gene encoding the proinflammatory chemokine, Cxcl10, a C-X-C motif cytokine involved in the development of T cell effector function. Five other immune response genes, mostly involved in regulating inflammatory immune responses and antigen processing, were present in this group of highly differentially expressed genes, together representing 48% of the 21 most highly differentially expressed genes (4T1 > 4T1ρ^0^). The only gene in this group of immune response genes that has been previously identified as being differentially expressed between wild type and ρ^0^ tumor cells is *PSMB8* (Magda et al., 2008), but in this case, it was more highly expressed in A549ρ^0^ cells than in A549 human lung adenocarcinoma cells.

Our results have confirmed that 4T1 cells express transcripts for the monocyte-recruiting chemokines Ccl2 (MCP-1) and Ccl5 (RANTES) as shown previously in 4T1 cells (Kurt et al., 2001; Vitiello et al., 2004; DuPré et al., 2007) and 4T1 cells isolated from 4T1 tumors (DuPré et al., 2007), with both chemokines present in conditioned media from 4T1 cells (Kurt et al., 2001; Vitiello et al., 2004; Cho et al., 2012; Kuan and Ziegler, 2018). Ccl2 in conditioned media from 4T1 cells was effective in amplifying macrophage-mediated innate inflammatory immune responses that included Ccl2 production in the presence and absence of the lipopolysaccharide (LPS) and migration (Cho et al., 2012; Madera et al., 2015), as well as phagocytosis (Madera et al., 2015).

Stress response-related genes were also well represented in the 21 most highly expressed genes in 4T1 cells compared with 4T1ρ^0^ cells, with five genes in this group. Of particular interest is Serpinf1 (pigment epithelial-derived factor, PEDF), a member of the large serum proteinase inhibitor family that lacks proteinase activity and is a potent inhibitor of angiogenesis and adipogenesis *via* PPAR-γ (Gattu et al., 2013). Serpinf1 promotes inflammation; impairs glucose uptake, ATP production, and mitochondrial function; and compromises fatty acid oxidation, while increasing the expression of genes involved in fatty acid oxidation *via* PPAR-γ (Carnagarin et al., 2015). Other genes in this group include those encoding the glutathione-S-transferases, *Gstk1* and *Gstm1*, the cysteine proteinase inhibitor, *Cst6*, and the cysteine- and glycine-rich protein-2, *Csrp2*, that is down-regulated in response to cell injury.

Notably, 12 of 15 (80%) of the most highly differentially expressed immune and stress response-related genes expressed in 4T1 cells recorded zero reads in 4T1ρ^0^ cells. In this context, zero reads by MinION nanopore cDNA sequencing is a highly relevant result and a major point of difference between this 3rd generation sequencing platform as applied to gene expression analysis and other 2rd generation sequencing methodologies, all of which are insensitive to low expressing genes because of high background noise. Overall, 17 genes with log_2_FC > 4 (16-fold) showed no transcripts in 4T1ρ^0^ cells, whereas 29 of 40 genes with log_2_FC ≥ 2.0 were not expressed. These results were unexpected and show the potential of MinION nanopore cDNA sequencing to identify highly significant differential expression of nuclear genes that are not expressed in ρ^0^ cells and therefore require the presence of mtDNA for their expression.

We also showed that the expression of most of the immune and stress response genes whose expression was compromised in 4T1ρ^0^ cells partially or fully recovered expression in 4T1ρ^0^ SC cells that had acquired mitochondria, and this was confirmed by qRT-PCR analysis for a subset of these genes. A notable exception was *Serpinf1* where little recovery of gene expression was observed, suggesting a more complex mechanism of regulatory control for this gene.

A role for mitochondrial respiration in regulating the expression of nuclear genes involved in tumor immune responses has not been described before. Depending on the activation status of immune cells, both anti-tumor (type 1) and tumor-supporting (type 2) responses have been documented. With respect to infiltration of immune cells into the early breast tumor microenvironment, macrophages are the main players, constituting up to 50% of the tumor mass in these tumors (Obeid et al., 2013). Macrophages readily switch between M1 anti-tumor and M2 tumor-supporting phenotypes (Obeid et al., 2013). With respect to tumor progression, tumor-associated macrophages (TAMs) have been associated with poor patient survival (Bingle et al., 2002). We show here that macrophage recruitment was extremely low in 4T1ρ^0^ tumors 1 day after injection, but that recruitment normalized to parental levels by Day 3, and that the recruited cells were likely of the M2 phenotype (F4/80^pos^ and FAP^pos^, Figure 7b). Interestingly, mtDNA was present in Day 4 tumors and may have been present before that time point at levels below the detection limit of our methodology. Our *in vitro* work in co-cultures has shown that mitochondrial transfer occurs within 24 h of co-culture in a small percentage of cells (unpublished data). In addition to 4T1 cells, cancer-associated fibroblasts (CAFs) also release Ccl2 and Ccl5 to recruit monocytes/macrophages and re-polarize M1 macrophages to M2-like TAMs (Soria and Ben-Baruch, 2000; Owen et al., 2011; Biswas et al., 2014; Katanov et al., 2015; Madera et al., 2015; Córdova et al., 2017; Liubomirski et al., 2019). Thus, it is possible that increased macrophage recruitment by Day 3 may involve Ccl2 release by CAFs at a very early stage of tumor formation. We further found that a delay in macrophage recruitment did not affect tumor progression as the 4T1ρ^0^ SC tumors remained very small until Day 21 when they became palpable and when the increase in tumor mass was obvious (Bajzikova et al., 2019).

Our results have shown that mitochondrially encoded gene expression was restored to normal levels after mitochondrial acquisition. Interestingly, the expression of most nuclear genes that encode subunits of mitochondrial respiratory complexes and mitochondrial ribosomal proteins was not markedly affected by loss of mtDNA. For other nuclear genes whose expression was affected, the expression was partially or fully restored following mitochondrial acquisition. We were unable to identify nuclear genes that might be involved in recruitment of cells known to act as mitochondrial donors (Spees et al., 2006; Herst et al., 2018a). However, we did identify lack of expression of immune-related genes involved in monocyte/macrophage recruitment and lack of recruitment of these cells into the 4T1ρ^0^ cell mass on Day 1.

Although the mechanism(s) whereby the presence or absence of mtDNA affects the regulation of nuclear expression is unclear, our results lead us to conclude that mitochondrial acquisition by tumor cells without mtDNA results in bioenergetic remodeling and re-expression of genes involved in immune function and stress adaptation.

## Data Availability Statement

The datasets presented in this study can be found in online repositories. The names of the repository/repositories and accession number(s) can be found below: https://www.ebi.ac.uk/ena, https://www.ebi.ac.uk/ena/data/view/PRJEB35129, https://doi.org/10.5281/zenodo.1244087, https://doi.org/10.5281/zenodo.1244087, https://doi.org/10.17504/protocols.io.8azhsf6, and https://doi.org/10.17504/protocols.io.8azhsf6.

## Ethics Statement

The animal study was reviewed and approved by the Victoria University of Wellington Animal Ethics Committee.

## Author Contributions

MB, PH, and DE designed the research. CG, DE, PH, M-SF, SB, and RD conducted the research. MB, PH, and CG wrote the manuscript. All authors contributed to manuscript revision.

## Conflict of Interest

The authors declare that the research was conducted in the absence of any commercial or financial relationships that could be construed as a potential conflict of interest.
