# Supplementary material for: Mitochondrial DNA Affects the Expression of Nuclear Genes Involved in Immune and Stress Responses in a Breast Cancer Model
Source: Front Physiol. 2020 Nov 24;11:543962. doi: 10.3389/fphys.2020.543962 (PMC7732479; doi:10.3389/fphys.2020.543962)
Supplement: Supplementary file 1 [file Table_1.DOCX]

SUPPLEMENTARY TABLE T1:

| **TOP 40 GENES EXPRESSED HIGHER IN 4T1** | | | | | | | | | | | | |
| --- | --- | --- | --- | --- | --- | --- | --- | --- | --- | --- | --- | --- |
| **No.** | **Gene** | **Gene Description** | **Raw data 4T1**ρ0 **(A)** | **Raw data 4T1**ρ0 **(B)** | **Raw data 4T1**ρ0 **(C)** | **Raw data 4T1 (A)** | **Raw data 4T1 (B)** | **Raw data 4T1 (B)** | **Raw data 4T1 (D)** | **Log_2_ Fold Change (4T1 vs 4T1**ρ0**)** | **Log_2_ Fold Change SE** | ***p* value adjusted** |
| 1 | **Ccl2** | C-C motif chemokine 2 | 0 | 0 | 0 | 641 | 99 | 139 | 14 | **9.9** | 1.7 | 1.4E-07 |
| 2 | **Psmb8** | Proteasome subunit beta type-8 | 0 | 0 | 0 | 40 | 42 | 169 | 34 | **8.1** | 1.6 | 8.2E-06 |
| 3 | **Serpinf1** | Pigment epithelium-derived factor | 0 | 0 | 0 | 27 | 68 | 31 | 63 | **7.7** | 1.3 | 6.5E-08 |
| 4 | **Gstk1** | Glutathione S-transferase kappa 1 | 0 | 0 | 0 | 15 | 118 | 39 | 19 | **7.4** | 1.5 | 7.1E-06 |
| 5 | **Sumo3** | small ubiquitin-like modifier 3 | 0 | 0 | 0 | 32 | 22 | 35 | 18 | **7.2** | 1.3 | 5.5E-07 |
| 6 | **Nme4** | Nucleoside diphosphate kinase, mitochondrial | 0 | 0 | 0 | 6 | 35 | 15 | 25 | **6.4** | 1.4 | 4.2E-05 |
| 7 | **Tst** | thiosulfate sulfurtransferase, mitochondrial | 0 | 0 | 0 | 9 | 15 | 17 | 24 | **6.3** | 1.3 | 3.8E-05 |
| 8 | **Ptpn18** | protein tyrosine phosphatase, non-receptor type 18 | 0 | 0 | 0 | 7 | 30 | 20 | 10 | **6.2** | 1.4 | 7.6E-05 |
| 9 | **Smpdl3b** | sphingomyelin phosphodiesterase, acid-like 3B | 0 | 0 | 0 | 9 | 9 | 31 | 11 | **6.1** | 1.5 | 1.6E-04 |
| 10 | **Cxcl10** | C-X-C motif chemokine 10 | 0 | 0 | 0 | 3 | 1 | 54 | 16 | **6.1** | 1.8 | 7.2E-04 |
| 11 | **Rgs10** | regulator of G-protein signalling 10 | 0 | 0 | 0 | 9 | 29 | 14 | 7 | **6.0** | 1.5 | 1.8E-04 |
| 12 | **Gstm1** | Glutathione S-transferase Mu 1 | 0 | 0 | 0 | 10 | 20 | 14 | 7 | **5.7** | 1.7 | 7.3E-04 |
| 13 | **Glipr2** | Golgi-associated plant pathosis-related protein 1 | 0 | 0 | 0 | 2 | 19 | 12 | 15 | **5.5** | 1.6 | 7.1E-04 |
| 14 | **Ggh** | Gamma-glutamyl hydrolase | 0 | 0 | 0 | 3 | 23 | 8 | 12 | **5.4** | 1.7 | 1.0E-03 |
| 15 | **Ccl5** | C-C motif chemokine 5 | 2 | 3 | 8 | 31 | 70 | 250 | 54 | **4.8** | 0.7 | 9.0E-09 |
| 16 | **Map1lc3a** | Microtubule-associated proteins 1A/1B light chain ... | 0 | 0 | 0 | 3 | 15 | 6 | 9 | **4.7** | 1.9 | 2.8E-03 |
| 17 | **Gng11** | guanine nucleotide binding protein (G protein), ga... | 2 | 4 | 1 | 15 | 112 | 54 | 38 | **4.5** | 0.9 | 3.2E-06 |
| 18 | **Mgp** | matrix Gla protein | 0 | 2 | 0 | 7 | 231 | 55 | 56 | **4.5** | 3.1 | 1.2E-02 |
| 19 | **Cst6** | cystatin E/M | 0 | 4 | 3 | 15 | 97 | 37 | 45 | **4.4** | 1.0 | 2.3E-05 |
| 20 | **Psmb9** | proteasome (prosome, macropain) subunit, beta type... | 0 | 3 | 0 | 6 | 22 | 72 | 7 | **4.2** | 1.8 | 3.0E-03 |
| 21 | **Csrp2** | Cysteine and glycine-rich protein 2 | 0 | 0 | 0 | 2 | 13 | 7 | 3 | **4.1** | 2.0 | 4.9E-03 |
| 22 | **Tap2** | transporter 2, ATP-binding cassette, sub-family B ... | 0 | 0 | 0 | 7 | 5 | 11 | 4 | **3.9** | 2.3 | 8.5E-03 |
| 23 | **Anxa6** | Annexin A6 | 0 | 0 | 0 | 5 | 4 | 13 | 2 | **3.7** | 2.3 | 9.2E-03 |
| 24 | **Tnnc1** | Troponin C, slow skeletal and cardiac muscles | 3 | 1 | 2 | 1 | 106 | 19 | 55 | **3.7** | 1.4 | 1.6E-03 |
| 25 | **Nadk** | NAD kinase | 0 | 0 | 0 | 3 | 9 | 9 | 5 | **3.6** | 2.3 | 1.1E-02 |
| 26 | **Fcgrt** | Mus musculus Fc receptor, IgG, alpha chain transpo... | 0 | 0 | 0 | 7 | 7 | 2 | 13 | **3.5** | 2.5 | 1.4E-02 |
| 27 | **Tcea3** | Transcription elongation factor A protein 3 | 0 | 0 | 0 | 1 | 10 | 11 | 3 | **3.3** | 2.3 | 1.4E-02 |
| 28 | **Tle6** | transducin-like enhancer of split 6 | 0 | 0 | 0 | 2 | 6 | 8 | 6 | **3.1** | 2.3 | 1.6E-02 |
| 29 | **Syne4** | Nesprin-4 | 2 | 1 | 1 | 5 | 22 | 12 | 30 | **3.1** | 1.4 | 2.8E-03 |
| 30 | **Fbxo2** | F-box only protein 2 | 3 | 2 | 0 | 13 | 18 | 18 | 15 | **2.8** | 1.2 | 2.4E-03 |
| 31 | **Sord** | sorbitol dehydrogenase | 0 | 0 | 0 | 5 | 7 | 3 | 4 | **2.8** | 2.3 | 2.0E-02 |
| 32 | **Atp1b1** | Sodium/potassium-transporting ATPase subunit beta-... | 0 | 0 | 0 | 6 | 9 | 5 | 2 | **2.7** | 2.4 | 2.3E-02 |
| 33 | **Cryl1** | Lambda-crystallin homolog | 0 | 3 | 0 | 7 | 33 | 14 | 5 | **2.7** | 1.8 | 1.1E-02 |
| 34 | **Gyg** | glycogenin | 1 | 5 | 3 | 6 | 23 | 27 | 29 | **2.6** | 0.9 | 5.7E-04 |
| 35 | **Selenom** | Selenoprotein M | 5 | 23 | 12 | 46 | 149 | 64 | 49 | **2.3** | 0.8 | 6.8E-04 |
| 36 | **Hpcal1** | Hippocalcin-like protein 1 | 1 | 1 | 1 | 6 | 27 | 20 | 6 | **2.3** | 1.8 | 1.9E-02 |
| 37 | **Inpp1** | Inositol polyphosphate 1-phosphatase | 0 | 0 | 0 | 4 | 5 | 8 | 2 | **2.3** | 2.3 | 3.3E-02 |
| 38 | **Casp7** | Caspase-7 Caspase-7 subunit p20 Caspase-7 subunit ... | 0 | 0 | 0 | 4 | 3 | 6 | 2 | **2.2** | 2.1 | 2.9E-02 |
| 39 | **Crip2** | Cysteine-rich protein 2 | 0 | 0 | 0 | 2 | 9 | 5 | 2 | **2.1** | 2.1 | 3.5E-02 |
| 40 | **Agpat4** | 1-acyl-sn-glycerol-3-phosphate acyltransferase del... | 6 | 8 | 11 | 18 | 40 | 39 | 43 | **2.0** | 0.6 | 2.1E-04 |
