# Supplementary material for: Mitochondrial DNA Affects the Expression of Nuclear Genes Involved in Immune and Stress Responses in a Breast Cancer Model
Source: Front Physiol. 2020 Nov 24;11:543962. doi: 10.3389/fphys.2020.543962 (PMC7732479; doi:10.3389/fphys.2020.543962)
Supplement: Supplementary file 2 [file Table_2.DOCX]

SUPPLEMENTARY TABLE T2:

| **TOP 40 GENES EXPRESSED HIGHER IN 4T1** ρ0 | | | | | | | | | | | | |
| --- | --- | --- | --- | --- | --- | --- | --- | --- | --- | --- | --- | --- |
| **No.** | **Gene** | **Gene Description** | **Raw data 4T1**ρ0 **(A)** | **Raw data 4T1**ρ0 **(B)** | **Raw data 4T1**ρ0 **(C)** | **Raw data 4T1 (A)** | **Raw data 4T1 (B)** | **Raw data 4T1 (B)** | **Raw data 4T1 (D)** | **Log_2_ Fold Change (4T1 vs 4T1**ρ0 **)** | **Log_2_ Fold Change SE** | ***p* value adjusted** |
| 1 | **Krt7** | Keratin, type II cytoskeletal 7 | 551 | 546 | 806 | 0 | 1 | 0 | 0 | **-9.4** | 1.9 | 4.6E-06 |
| 2 | **Sfn** | stratifin | 64 | 109 | 329 | 0 | 0 | 0 | 0 | **-8.9** | 1.2 | 1.3E-11 |
| 3 | **Dynap** | Dynactin Associated Protein | 39 | 92 | 62 | 0 | 0 | 0 | 0 | **-7.6** | 1.2 | 7.4E-09 |
| 4 | **Mal** | Myelin and lymphocyte protein | 52 | 108 | 34 | 0 | 0 | 0 | 0 | **-7.5** | 1.3 | 2.6E-07 |
| 5 | **Cldn6** | Claudin-6 | 39 | 165 | 119 | 0 | 1 | 0 | 0 | **-7.5** | 1.2 | 1.5E-08 |
| 6 | **Perp** | p53 apoptosis effector related to PMP-22 | 40 | 56 | 57 | 0 | 0 | 0 | 0 | **-7.3** | 1.2 | 1.8E-08 |
| 7 | **Sox15** | SRY (sex determining region Y)-box 15 | 24 | 58 | 43 | 0 | 0 | 0 | 0 | **-7.0** | 1.1 | 3.8E-08 |
| 8 | **Ice2** | Little elongation complex subunit 2 | 82 | 241 | 320 | 0 | 4 | 2 | 0 | **-6.6** | 0.8 | 7.2E-15 |
| 9 | **Tubb2a** | tubulin, beta 2A class IIA | 20 | 43 | 88 | 1 | 0 | 0 | 0 | **-6.6** | 1.1 | 3.4E-07 |
| 10 | **Prl2c3** | Mus musculus prolactin family 2, subfamily c, memb... | 334 | 1090 | 1937 | 12 | 4 | 3 | 0 | **-6.5** | 1.4 | 3.8E-05 |
| 11 | **Sult2b1** | Sulfotransferase family cytosolic 2B member 1 | 13 | 52 | 27 | 0 | 0 | 0 | 0 | **-6.4** | 1.3 | 3.5E-05 |
| 12 | **Prss22** | protease, serine 22 | 38 | 65 | 74 | 1 | 0 | 0 | 0 | **-6.2** | 1.9 | 8.5E-04 |
| 13 | **Krt14** | Keratin, type I cytoskeletal 14 | 4 | 58 | 19 | 0 | 0 | 0 | 0 | **-5.2** | 2.3 | 3.9E-03 |
| 14 | **Bhlhe40** | Class E basic helix-loop-helix protein 40 | 7 | 19 | 37 | 2 | 0 | 0 | 0 | **-4.0** | 1.7 | 3.2E-03 |
| 15 | **Klk10** | kallikrein related peptidase 10 | 308 | 839 | 845 | 11 | 49 | 69 | 7 | **-3.8** | 0.7 | 4.5E-07 |
| 16 | **Tnfaip2** | tumor necrosis factor, alpha-induced protein 2 | 6 | 15 | 17 | 0 | 1 | 0 | 0 | **-3.7** | 1.9 | 5.8E-03 |
| 17 | **Nxf1** | Nuclear RNA export factor 1 | 54 | 174 | 109 | 5 | 6 | 5 | 10 | **-3.6** | 0.5 | 7.9E-14 |
| 18 | **Prl2c2** | Prolactin-2C2 | 245 | 442 | 739 | 10 | 0 | 2 | 0 | **-3.1** | 2.8 | 2.4E-02 |
| 19 | **Ctxn1** | Cortexin-1 | 14 | 30 | 25 | 1 | 4 | 1 | 1 | **-3.0** | 0.9 | 1.8E-04 |
| 20 | **Ift172** | Intraflagellar transport | 19 | 67 | 46 | 2 | 6 | 3 | 2 | **-2.9** | 0.9 | 3.6E-04 |
| 21 | **Hist2h2be** | Histone H2B type 2-E | 4 | 32 | 3 | 0 | 0 | 0 | 0 | **-2.8** | 2.5 | 2.5E-02 |
| 22 | **Mest** | Mesoderm-specific transcript protein | 5 | 7 | 7 | 0 | 0 | 0 | 0 | **-2.8** | 2.1 | 1.7E-02 |
| 23 | **Wnt7a** | wingless-type MMTV integration site family, member... | 9 | 7 | 3 | 0 | 0 | 0 | 0 | **-2.7** | 2.2 | 1.9E-02 |
| 24 | **Aig1** | Mus musculus androgen-induced 1 (Aig1), transcript... | 6 | 7 | 5 | 0 | 0 | 0 | 0 | **-2.5** | 2.1 | 2.3E-02 |
| 25 | **Crip1** | Cysteine-rich protein 1 | 1431 | 7680 | 4994 | 216 | 1273 | 360 | 65 | **-2.4** | 0.9 | 7.5E-04 |
| 26 | **Erg28** | Probable ergosterol biosynthetic protein 28 | 86 | 171 | 132 | 10 | 19 | 27 | 11 | **-2.4** | 0.5 | 4.5E-07 |
| 27 | **Cst13** | Cystatin-13 | 4 | 41 | 7 | 0 | 2 | 0 | 0 | **-2.3** | 2.1 | 2.6E-02 |
| 28 | **Rab32** | RAB32, member RAS oncogene family | 4 | 4 | 7 | 0 | 0 | 0 | 0 | **-2.2** | 2.0 | 2.4E-02 |
| 29 | **Msln** | Mus musculus mesothelin (Msln), transcript variant... | 100 | 223 | 134 | 7 | 29 | 24 | 35 | **-2.2** | 0.4 | 4.5E-07 |
| 30 | **Epb41l4a** | Band 4.1-like protein 4A | 19 | 24 | 28 | 3 | 6 | 2 | 1 | **-2.1** | 0.8 | 1.6E-03 |
| 31 | **Nppb** | natriuretic peptide type B | 13 | 13 | 34 | 1 | 1 | 2 | 0 | **-2.0** | 1.7 | 2.3E-02 |
| 32 | **Lat2** | Linker for activation of T-cells family member 2 | 16 | 44 | 32 | 1 | 4 | 4 | 2 | **-2.0** | 1.2 | 8.4E-03 |
| 33 | **Cbr1** | Carbonyl reductase | 11 | 13 | 14 | 0 | 0 | 1 | 2 | **-2.0** | 1.6 | 2.2E-02 |
| 34 | **Prelid2** | PRELI domain-containing protein 2 | 29 | 141 | 110 | 2 | 35 | 9 | 4 | **-1.9** | 0.9 | 3.3E-03 |
| 35 | **Mettl7b** | Methyltransferase-like protein 7B | 53 | 73 | 141 | 2 | 14 | 12 | 7 | **-1.7** | 1.2 | 1.8E-02 |
| 36 | **Reep5** | receptor accessory protein 5 | 171 | 478 | 341 | 45 | 106 | 66 | 71 | **-1.7** | 0.3 | 8.7E-08 |
| 37 | **Hmgb3** | High mobility group protein B3 | 3 | 5 | 6 | 0 | 0 | 0 | 0 | **-1.6** | 1.8 | 4.9E-02 |
| 38 | **Msmo1** | Methylsterol monooxygenase 1 | 62 | 122 | 118 | 17 | 23 | 15 | 28 | **-1.6** | 0.4 | 8.7E-05 |
| 39 | **Rbfa** | ribosome binding factor A | 5 | 9 | 0 | 0 | 0 | 0 | 0 | **-1.6** | 1.8 | 5.3E-02 |
| 40 | **Nsdhl** | Sterol-4-alpha-carboxylate 3-dehydrogenase, decarb... | 19 | 25 | 40 | 2 | 5 | 8 | 2 | **-1.6** | 0.81 | 8.7E-03 |
