# Supplementary figures and images for: Mitochondrial DNA Affects the Expression of Nuclear Genes Involved in Immune and Stress Responses in a Breast Cancer Model
Source: Front Physiol. 2020 Nov 24;11:543962. doi: 10.3389/fphys.2020.543962 (PMC7732479; doi:10.3389/fphys.2020.543962)

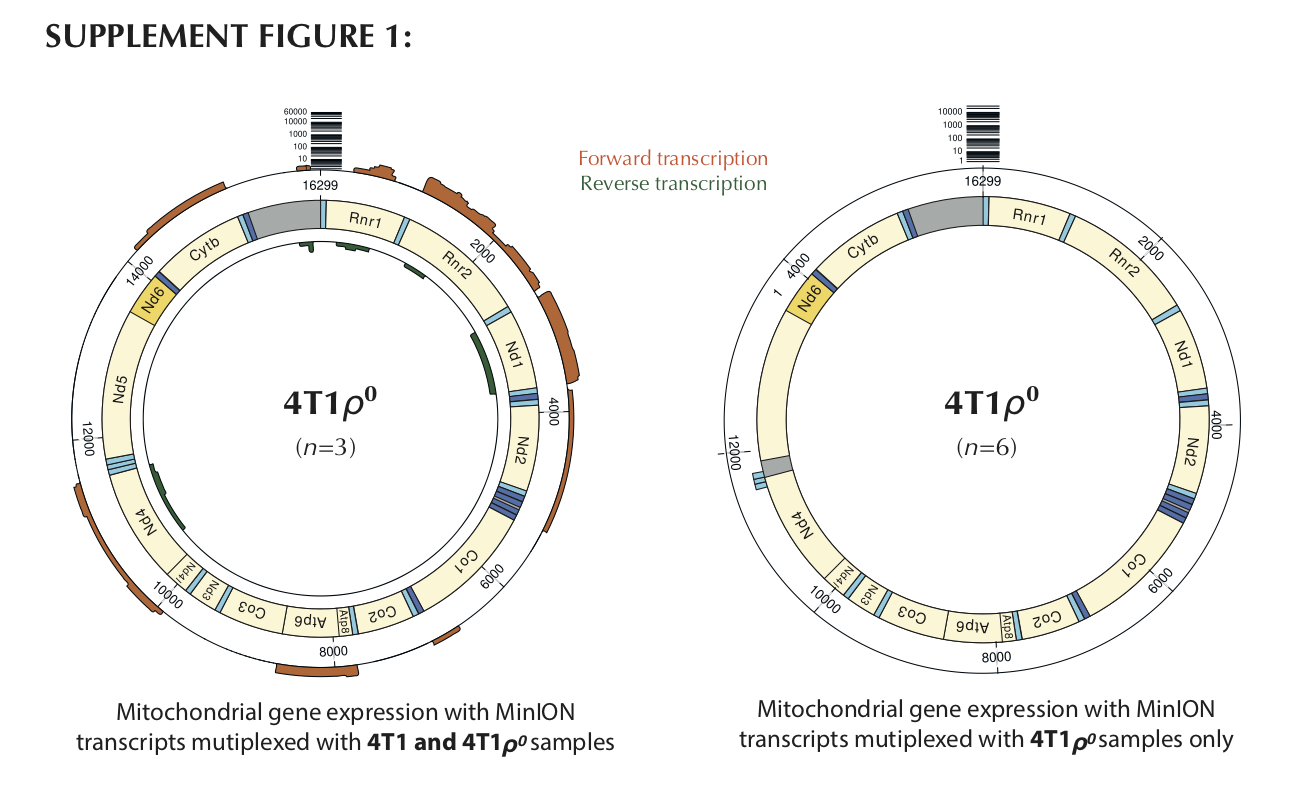

Supplement: Supplementary file 3 [file Image_1.TIFF]
